# Subterranean Termite Social Alarm and Hygienic Responses to Fungal Pathogens

**DOI:** 10.3390/insects10080240

**Published:** 2019-08-05

**Authors:** Mark S. Bulmer, Bruno A. Franco, Edith G. Fields

**Affiliations:** 1 Department of Biological Sciences, Towson University, 341 Smith Hall, 8000 York Rd., Towson, MD 21252, USA; 2 Towson High School, 69 Cedar Ave, Towson, MD 21286, USA

**Keywords:** social immunity, entomopathogens, alarm behavior, allogrooming, *Metarhizium*

## Abstract

In social insects, alerting nestmates to the presence of a pathogen should be critical for limiting its spread and initiating social mechanisms of defense. Here we show that subterranean termites use elevated vibratory alarm behavior to help prevent fatal fungal infections. The elevated alarm leads to elevated social hygiene. This requires that termites coalesce so that they can groom each other’s cuticular surfaces of contaminating conidial spores. Groups of 12 *Reticulitermes flavipes* workers varied in their response when immersed in conidia solutions of nine different strains of *Metarhizium*. Pathogen alarm displays of short 2–7-second bursts of rapid longitudinal oscillatory movement (LOM), observed over 12 min following a fungal challenge, were positively correlated with the time that workers spent aggregated together grooming each other. The frequency of these LOMs was inversely correlated with fatal fungal infections. The variation in fatalities appeared to be largely attributable to a differential response to *Metarhizium brunneum* and *Metarhizium robertsii* in the time spent in aggregations and the frequency of allogrooming. Isolated workers challenged with conidia did not display LOMs, which suggests that the alarm is a conditional social response. LOMs appear to help signal the presence of fungal pathogens whose virulence depends on the level of this emergency alert.

## 1. Introduction

An effective immune system requires a rapid response in order to curtail the replication and spread of a pathogen. This is especially true in social insect colonies in which frequent social contact exacerbates transmission of pathogenic propagules. A typical behavioral response in ants and termites is to increase allogrooming rates after they have been challenged with the conidia of entomopathogenic fungi such as *Metarhizium* and *Beauveria* [1,2]. The conidia are destroyed when grooming brings them in contact with antimicrobial secretions or when they are ingested. Groups are often better at surviving challenges with conidia than individuals apparently because allogrooming affords greater protection against infection than autogrooming (for example [3,4]). This frontline strategy is especially important in the defense against microparasites such as *Metarhizium*, which is commonly encountered by insects that move through the soil [5]. Its conidial spores attach and rapidly grow through the insect cuticle and, once inside the hemocoel, replicating fungal cells mask themselves from cellular and humoral defense mechanisms with the secretion of a collagenous coat [6]. This evasive strategy appears to be effective against termites, which are less susceptible to fatal infections when exposed to a mutant strain that cannot produce the coat [7].

Termites also show a distinct alarm behavior when exposed to *Metarhizium* conidia, which typically consists of a 2–7-s burst of a rapid longitudinal oscillatory movement (LOM) with tarsi attached to the substrate for the duration of the LOM ([8,9], this study). Interestingly, two quite different behavioral responses have been described with the onset of the parasite-induced alarm. Rosengaus et al. (1999) showed that, in the damp-wood termite *Zootermopsis angusticollis*, individuals exposed to high concentrations of *Metarhizium* conidia and displaying LOMs were avoided by unchallenged individuals [8]. Individuals that had not been exposed to the fungus were apparently using vibrations in the substrate as a warning to stay away from infected individuals. Myles (2002) also showed that, in the subterranean termite *Reticulitermes flavipes*, exposure to high concentrations of *Metarhizium* conidia resulted in elevated LOMs [9]. However, the alarm behavior was associated with the aggregation of termites, which possibly affords protection through increased allogrooming.

There are surprisingly few reports of termite alarm behavior in response to entomopathogens given the extensive investigation of *Metarhizium* for biological control. This may be due to the design of many virulence studies that do not include sustained observation after termites have been challenged with conidia [9]. There is typically a delay in the onset of alarm behavior after the fungal challenge, which peaks around 12–15 min. Another possibility is that the behavior is not easily triggered by the laboratory strains that have been employed in these studies because the termites are unfamiliar with them. *Metarhizium* species and strains that are found within the vicinity of colonies may be more readily recognized as a pathogenic threat that requires a rapid response.

Natural epizootics are rarely observed in termites [10]. However, studies of the effects of inbreeding and the evolution of antifungal peptides suggest that termites face strong selective pressure from fungal pathogens [11,12,13]. Colony genetic diversity may be important for the termite immune defense, which has been shown to be true in ants and social bees [14]. Inbred colonies of *Z. angusticollis* are more susceptible to infection by *Metarhizium anisopliae* and have higher loads of cuticular microbes than outbred colonies [11]. Further evidence is provided by molecular research indicating that the selection by fungal pathogens has favored beneficial modifications in defensin-like peptides (termicins) that protect termites from infection [12,15].

The termite salivary gland appears to be an important source of antifungal proteins. These proteins may, therefore, inactivate both ingested conidia and conidia that have become attached to the cuticle. In subterranean termites, allogrooming rates increase with an initial conidial challenge apparently to facilitate their removal by ingestion before they attach to the cuticle [3]. The rate increases again around 12 h post-challenge, which is when conidia have become firmly attached to the cuticle and start to germinate [16]. At this critical point, the external activity of antifungal proteins such as termicins and β-1,3-glucanases, that are disseminated by allogrooming, may still be effective at protecting the termites from internal infections [13]. Furthermore, β-1,3-glucanases degrade the fungal wall, which may release elicitors that are important in activating innate and behavioral mechanisms of defense.

Groups of *R. flavipes* workers vary in their susceptibility to fatal infections from different locally encountered *Metarhizium* strains [17], which did not appear to be due to the variability in the health of the termites or viability of the fungal spores used to challenge the insects. The variation in the effectors of immune defense systems may account for this variation in vulnerability to fatal infections. Recognition may also be key, as an effective immune strategy is only of value if it is quickly activated. Termites may differ in their response to different strains and species because they do not consistently employ their antifungal defenses with equal vigor, and the variability in response may be due to the variability in detecting fungal conidia. In this study, we investigated whether social behaviors in the first few minutes after exposure to fungal conidia could be important in protecting termites from fatal infections. We recorded the initial behavioral response of workers from two colonies of *R. flavipes* to nine strains of *Metarhizium*, and tested whether there is a correlation between alarm behavior, allogrooming, aggregation, and survivorship. 

## 2. Materials and Methods

### 2.1. Study Organisms

Foraging groups of *R. flavipes* (>1000) were collected from three colonies located in Monkton, Maryland (Towson University Field Station). The termites were found in decaying logs separated by at least 60 m, which is usually sufficient to ensure that collections are from different colonies [18]. Wood containing termites was cut into manageable lengths and stored in plastic containers under dark, moist conditions at 24 °C. Workers were gently isolated from wood pieces and maintained in groups of 12 in 55 mm diameter Petri dishes lined with moistened filter paper (Whatman 1) for 24 h in ambient light prior to being used for our experiments. This allowed them to acclimatize to the light and clean their cuticular surfaces of particulate debris prior to the fungal exposures.

Eight *Metarhizium* strains were isolated from soil samples from Maryland (Monkton), Virginia (Huntly), Massachusetts (Middlesex Fells), North Carolina (Schenck Forest in Raleigh), and Texas (Lake Bryan) using a baiting technique with *Tenebrio molitor* larvae described by Denier and Bulmer (2015) [17]. An additional *Metarhizium* strain was isolated from an infected cadaver of an *Archotermopsis wroughtoni* cadaver collected from Himachal Pradesh, India (Jibhi). The conidia and mycelia of each of the nine *Metarhizium* strains (3–4 mm^2^) were harvested for purification with a QIAGEN DNeasy Blood and Tissue kit (protocol for DNA purification from animal tissue). The 5’ region of translation elongation factor 1-alpha was PCR-amplified using PCR primers EF1T and EF2T [19]. These regions were Sanger-sequenced (Macrogen USA sequencing service) for species identification of each strain. Variation in the 5’ region of the EF1 sequence is especially useful for identifying the *Metarhizium* species [19].

### 2.2. Group Response to Nine Strains of Metarhizium

Conidia were harvested from clonal lines sporulating on potato dextrose agar that included 50 μg mL^−1^ ampicillin (PDA-AMP) and suspended in 0.1% Tween 80 to create stock suspensions of approximately 10^7^ conidia mL^−1^. Stock suspensions were diluted with sterile water to 10^6^ conidia ml^−1^. The conidia concentrations in the suspensions were determined with a hemocytometer [17]. Twelve workers were then placed in 1.5 mL tubes containing 500 μL of the dilutions and gently flicked by hand for 10 s. The workers were subsequently deposited onto filter paper to absorb excess fluid and immediately transferred to 55 mm diameter Petri dishes lined with moistened filter paper (Whatman 1) and video recorded for 12 min using the camera on an iPhone 8 clamped to a laboratory stand. The mortality was monitored for 14 days. Deceased termites were removed, surface sterilized with 70% ethanol, and placed on moist filter paper in Petri dishes (55 mm) maintained at 24 °C and 100% humidity for confirmation of *Metarhizium* infection. This assay was repeated with nine *Metarhizium* strains with workers from two different colonies (two groups of 12 for each colony) and included controls in which termites were challenged with sterile water alone (*n* = 480 workers). Workers used in this assay were consistently sampled from the same two colonies. One control of sham-challenged termites was performed alongside each experimental exposure (*n* = 4) to the nine fungal strains.

The number of bouts of alarm (LOMs) was scored over the 12-min observation period. Each discrete LOM typically lasted between 2–7 s. For individual termites, LOMs had to have completely stopped and then resumed to be scored as separate events. Allogrooming events were scored when mouth to body contact between two termites involved the movement of mouthparts (palps). Discrete allogrooming events required paired termites to break contact and form new contacts to be recorded as separate events. Contact frequently occurred between termites that did not include allogrooming. To help control for error in recording allogrooming events, the observer was blind to the treatment category (controls and fungal strain), and allogrooming events were scored three times for each recording. An average of the three scores for each treatment was used in subsequent analysis. Aggregation was measured by recording the total time over the 12-min observation period that at least nine workers were touching or within 2 mm of each other throughout the dish in an unbroken sequence. Major body parts (head, thorax, and abdomen but not legs or antennae) had to be within 2 mm of each other to be considered contiguously connected.

### 2.3. The Isolated Individual Worker Response to Metarhizium

Groups of 12 workers were treated as described above with the *Metarhizium* strain (10^6^ conidia ml^−1^) that induced the highest levels of alarm among the nine different strains of fungus (strain LB12). Isolated individuals (*n* = 12), pairs (*n* = 6), and a single group of 12 workers were immediately separated into Petri dishes (35 mm diameter dishes for individuals and pairs, 55 mm for the group of 12) lined with moistened filter paper and video recorded for 12 min.

### 2.4. Group Response to a Sporulating Cadaver

**Figure 1:**
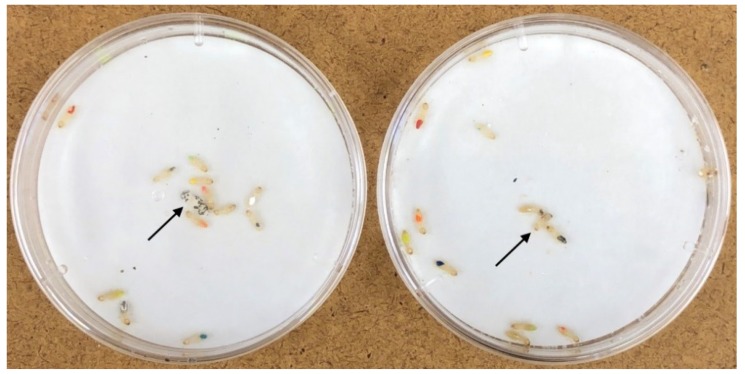
Workers exposed to a sporulating or control cadaver. For the left-hand side dish, arrow points to the sporulating cadaver. For the right-hand side dish, arrow points to the control cadaver (https://zenodo.org/record/3240589#.XPltDdNKgW8).

A group of 12 workers was presented with either a sporulating cadaver (killed by infection with *M. robertsii*, strain LB12) or a freshly killed cadaver as a control that was placed in the middle of a 55 mm dish lined with moistened filter paper. The control was killed by cold exposure, −20 °C for 5 min, and then warmed at RT for 10 min prior to being presented to the workers. The first 6 min of the 12-min video recordings were used for analysis of the order of contact with the cadaver and subsequent alarm displays. Individuals were marked with a dot of one of 12 different colors of enamel paint (Testors^®^, Vernon Hills, IL, USA) using a wooden toothpick to apply the paint to tergites of the abdomen (Figure 1). The paint was allowed to dry, and termites acclimatize for 24 h prior to being exposed to the sporulating or control cadaver.

### 2.5. Collective Humoral Defenses

Nine groups of 12 workers from one colony were exposed to the nine strains of *Metarhizium* or water for a control (*n* = 10, nine treatments and one control group) and LOMs were recorded as described above. Twenty-four hours after being immersed in conidia, the ten groups of 12 workers were chilled on ice and each group of cold-immobilized workers was homogenized in 60 μL sterile water with a Biomasher column (pore size 80–145 μm; VWR, Radnor, PA, USA) and then filter sterilized with 0.22 μm Ultrafree filters (MilliporeSigma, Burlington, MA, USA). Ten μL of the resulting crude extract or sterile water for the control was incubated with 10 μL of 10^4^ *M. guizhouense* conidia mL^−1^ and 20 μL of 200 μg mL^−1^ ampicillin (40 μL total volume) for 24 h. This *M. guizhouense* strain (MD335) was not one of the nine strains used to challenge termites. Two replicates of each crude extract mix and control were then plated on PDA-AMP and incubated at RT for four days. Conidia colony-forming units (CFUs) for the *Metarhizium* exposure treatments and the controls were averaged over the two replicates for subsequent analysis.

### 2.6. Group Response to Different Concentrations of Conidia 

The alarm response of groups of 12 workers to different concentrations of two strains of *Metarhizium* was investigated. Termites were immersed in 10^6^, 10^5^, 10^4^, 10^3^, and 0 conidia mL^−1^ and video recorded as described above. 

### 2.7. Statistical Analysis

We used workers from the same two colonies for the group response to the nine strains of *Metarhizium* and the behavioral observations were combined from two experiments for a single score for each colony (two colony replicates for each *Metarhizium* strain). The pooled data for alarm, mortality, aggregation, and allogrooming were normally distributed and analyzed with partial correlations and linear regression that controlled for colony effects (the partial correlation tests were two-tailed, and the regression residuals were normally and equally distributed in plots checking these assumptions). The plots of the measures revealed that aggregation scores increased exponentially and death, allogrooming, and alarm increased linearly. The aggregation scores were, therefore, log-transformed. The combined behavioral responses for each colony for four of the nine *Metarhizium* strains identified as *M. brunneum* were compared to four *Metarhizium* strains identified as *M. roberstii* with a *t*-test (two-tailed).

Termite mortality over 14 days was analzed with a Cox proportional-hazards regression model. Hazard ratios of death (HRs) for each *Metarhizium* strain were calculated from the regression analysis of termite survivorship by comparing the survivorship of termites from each colony treated with *Metarhizium* conidia to controls that were not exposed to conidia. This generated 18 HR scores that were used for partial correlation and regression analysis. HRs that controlled for colony effects for four of the nine *Metarhizium* strains identified as *M. brunneum* were compared to four *Metarhizium* strains identified as *M. roberstii* with a *t*-test (two-tailed).

For the group response to a sporulating or control cadaver, the number of LOMs for each individual over 12 min were compared between the sporulating and control treatment. This data was not normally distributed, and the two treatments were compared with a Mann–Whitney *U* test (two-tailed). To investigate whether contact with conidia was important for eliciting a behavioral response, LOMs between two categories of workers, the first six and last six to encounter the cadaver over 6 min, were compared to an expectation of an equal frequency of alarm irrespective of the contact order with a Chi-square test of homogeneity.

For the collective humoral defenses, a *Z*-test was used to determine if there was a significant correlation between LOMs and CFUs across the fungal treatments. The CFUs for four of the nine *Metarhizium* strains, identified as *M. brunneum,* were compared to four *Metarhizium* strains identified as *M. roberstii* with a *t*-test (two-tailed).

## 3. Results

### 3.1. Group Response to Nine Strains of Metarhizium

**Figure 2:**
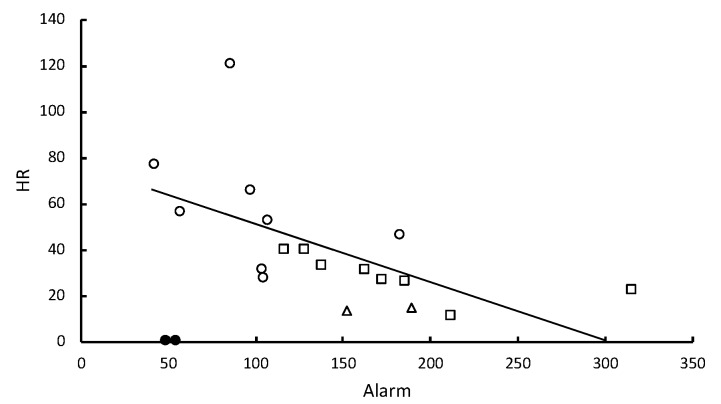
Hazard ratios of death relative to controls are predicted by the frequency of the pathogen alarm behavior (LOMs). Groups of 12 workers were challenged by brief immersion in the conidial suspensions of nine different strains of *Metarhizium* (for two colonies, *n* = 18). The strains corresponded with three different species. Open circles, *M. brunneum*; open squares, *M. roberstsii*; open triangles, *M. guizhouense*; solid circles, controls. The line of regression does not include controls. After controlling for colony effects, standardized β = −0.609, *p* = 0.008.

**Figure 3:**
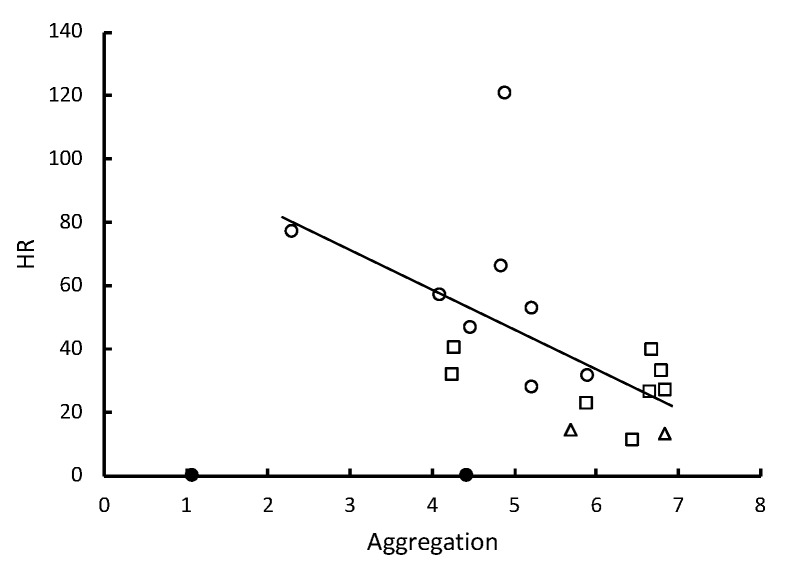
Hazard ratios of death relative to controls are predicted by the time workers spent in aggregations. Groups of 12 workers were challenged by brief immersion in the conidial suspensions of nine different strains of *Metarhizium* (for two colonies, *n* = 18). The strains corresponded with three different species. Open circles, *M. brunneum*; open squares, *M. roberstsii*; open triangles, *M. guizhouense*; solid circles, controls. The line of regression does not include controls. The time in seconds that workers spent in aggregations of greater than eight was log-transformed. After controlling for colony effects, standardized β = −0.580, *p* = 0.013.

**Figure 4:**
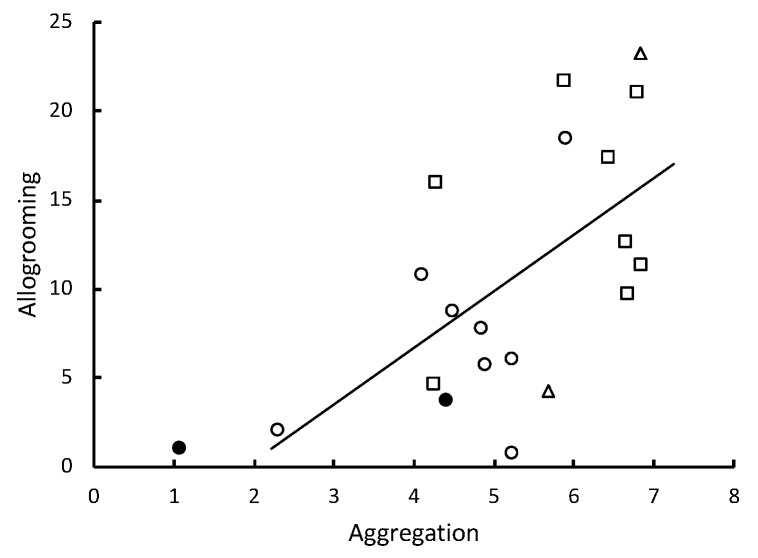
Increased allogrooming is predicted by the time workers spent in aggregations. Groups of 12 workers were challenged by brief immersion in the conidial suspensions of nine different strains of *Metarhizium* (for two colonies, *n* = 18). The strains corresponded with three different species. Open circles, *M. brunneum*; open squares, *M. roberstsii*; open triangles, *M. guizhouense*; solid circles, controls. The line of regression does not include controls. The time in seconds spent in aggregations was log-transformed. After controlling for colony effects, standardized β = 0.615, *p* = 0.003.

The hazard ratio of death (HR) due to infection with the nine different strains of *Metarhizium* showed a significant inverse correlation with the level of pathogen alarm behavior observed in the first 12 min following fungal challenges (Table 1, Figure 2). Almost all death was attributable to *Metarhizium* infection (99.1% confirmation). This alarm response indicates that *R. flavipes* workers vary in their ability to detect the different strains. The inverse correlation between HR and the number of allogrooming bouts was not significant (Table 1). However, the inverse correlation between HR and the time spent in aggregations was significant (Table 1, Figure 3) and allogrooming was significantly positively correlated with the time spent in aggregations (Table 1, Figure 4). Alarm behavior also showed a significant positive correlation with the observed number of allogrooming bouts in the first 12 min post-challenge (Table 1). The HRs for the nine different strains of *Metarhizium*, while controlling for colony effects, were all significantly different from controls (Table S1). 

### 3.2. Metarhizium Species Identity and Interspecific Behavioral Response

Four of the nine *Metarhizium* isolates were identified as *M. brunneum*, four as *M. robertsii*, and one as *M. guizhouense.* The *t*-tests indicated that *M. brunneum* elicited significantly weaker behavioral responses in the workers than *M. roberstsii* (for LOM’s, means = 195.3 and 357.0 respectively, *t* = −2.511, *p* < 0.05; for aggregation, means = 292.0 and 1129.5 respectively, *t* = −3.274, *p* < 0.05; for allogrooming, means = 14.9 and 28.6, respectively, *t* = −2.448, *p* < 0.05) and significantly higher HRs (means = 55.6 and 28.8, respectively, *t* = 2.801, *p* < 0.05). Cox regression also indicated a significantly higher risk of death after exposure to *M. brunneum* than *M. robertsii* conidia (1.9 times higher HR, Wald = 20.8, *p* < 0.001). 

### 3.3. Isolated Individual Worker Response to Metarhizium

Individual workers that were exposed to the *Metarhizium* strain (10^6^ conidia ml^−1^ of strain LB12) that induced the highest levels of alarm among the nine different strains of fungus (Table S1) showed no alarm behavior over 12 min of observation. A total of 23 LOM’s were observed in the six pairs of workers, and 115 LOMs were observed in the group of 12 workers. After one week there was 100% mortality for individuals, 66.7% for pairs and 25% for the group of 12 workers.

### 3.4. Group Response to a Sporulating Cadaver

Workers frequently came into contact and showed no avoidance of the sporulating cadaver. Workers also did not avoid the control cadaver. However, in contrast to the sporulating cadaver, there was some manipulation of the control cadaver with the mouthparts (allogrooming and biting). Over 12 min of observation, the number of LOMs for each individual in response to the sporulating cadaver was significantly greater than for the control cadaver (*n* = 105 versus 26 total LOMs for treatment and control, respectively, *U* = 26, *Z* = 2.627, *p* < 0.01). 

The contact with the sporulating cadaver appeared to be necessary for eliciting social behaviors. The onset of the alarm corresponded with the order of contact over the first six minutes of observation. For the sporulating cadaver treatment, the first six workers to come into contact with the cadaver showed significantly more alarm (*n* = 79 LOMs) than the remaining six (n = 26 LOMs) compared to an expectation of an equal frequency of alarm irrespective of the contact order (Chi-square = 26.75, *p* < 0.001). For the control cadaver, the first six workers to come into contact with the cadaver did not show a significant difference in the alarm (*n* = 13 LOMs) than the remaining six (n = 13 LOMs) compared to an expectation of an equal frequency of alarm irrespective of the contact order (Chi-square = 0, *p* = 1).

### 3.5. Collective Humoral Defenses

There was no significant correlation between the survival of conidia treated with crude extracts of termites (CFUs) that had been challenged with the nine strains of *Metarhizium* and their alarm response to these strains (*r* = −0.200, *Z* = −0.497, *p* = 0.619). There was also no significant difference between the CFUs after treatment with crude extracts of termites that had been challenged with either *M. brunnuem* or *M.robertsii* (CFU mean = 9.88 and 9.75, respectively, *t* = 0.033, *p* = 0.487). There were 21.5 CFUs (the average over two replicates) resulting from incubating the termite extract from the termites that had not been challenged with conidia. There were 63 CFUs (the average over two replicates) for the control conidia that were not exposed to crude extracts.

### 3.6. Group Response to Different Concentrations of Conidia

**Figure 5:**
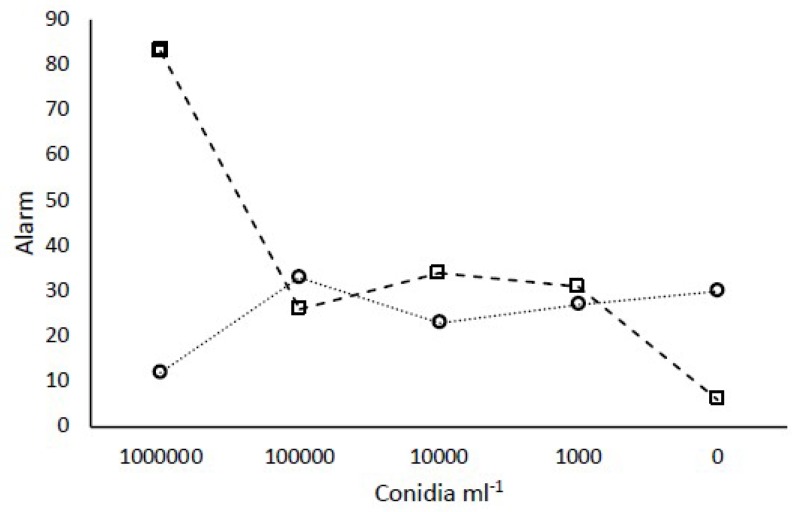
The frequency of the alarm displays (LOMs) in response to different concentrations of conidia of *M. brunneum* (open circles) or *M. robertsii* (open squares).

The two strains used to investigate concentration effects of conidia represented *M. robertsii* and *M. brunneum* (LB15 and MD002, respectively). Consistent with previous results, 10^6^ conidia mL^−1^ of *M. robertsii*, but not *M. brunneum*, elicited an elevated alarm above that seen for controls (Figure 5). Concentrations below 10^6^ conidia mL^−1^ for either fungal species did not elicit an elevated alarm. 

## 4. Discussion

One to two minutes after *R. flavipes* workers were briefly immersed in a conidial suspension or after they came into contact with a sporulating cadaver, they displayed an increased frequency of 2–7-second bouts of a rapid longitudinal oscillatory movement (pathogen alarm behavior or LOMs) relative to control treatments. The frequency of these bouts after first contact with conidia predicted survivorship of the groups of *R. flavipes* workers (Figure 2). The risk of fatalities after the groups of workers were challenged with the nine different strains of *Metarhizium* showed a significant inverse correlation with the level of pathogen alarm behavior observed in only the first 12 min after the fungal challenge. When the collective alarm response was sufficiently robust, workers aggregated and their frequency of allogrooming substantially increased (Figure 4). The workers appear to vary in their ability to recognize and rapidly respond to different strains of *Metarhizium*. A weaker alarm response leads to higher mortality. Some strains elicit a strong response, and others elicit a weak response similar to the response seen in controls immersed in water lacking conidia. 

Isolated workers challenged with conidia did not display LOMs, which suggests that the alarm is a conditional social response rather than an involuntary individual response to being contaminated with conidia. The worker pairs displayed LOMs but not consistently, possibly because individuals vary in their thresholds for recognizing and responding to a challenge. However, in groups of 12, the first workers to come into contact with a sporulating cadaver were the first to show an alarm response. The delay between the contact with conidia and the onset of alarm suggests that workers were unable to rapidly detect conidia by airborne odors or even with contact by antennation. Termites have been shown to recognize entomopathogenic fungi by using odors [20,21]; however, antennae may not be critical for recognition [22]. Our results indicate that chemoreception or mechanoreception by mouthparts rather than antennae was critical for recognition. Conidia detection that leads to allogrooming may frequently depend on the contact between two termites because it involves investigation of the cuticle with mouthparts.

The elevated alarm appeared to trigger aggregation and allogrooming. With exposure to a sufficient concentration of conidia, recognition of the pathogen, demonstrated by elevated LOMs, was followed by termites slowing their movement and aggregating so that they could apparently clean each other of conidia that had attached to their cuticular surfaces. After pathogen detection, the release of alarm pheromones may have also contributed to this response, although alarm pheromones are usually associated with an increase in motion and not the decrease that we observed [23]. The observed correlation between HRs and the number of allogrooming bouts was not significant for the comparison of different strains of *Metarhizium*; however, our 12 min snapshots of the initial response to infection missed a substantial amount of elevated allogrooming that occurs over a 24-h period and that is clearly critical for protection [16]. Moreover, alarm and allogrooming over the first 12 min were significantly correlated and reflected significant differences in virulence between *M. brunneum* and *M. robertsii*.

There was no correlation between the frequency of LOMs and the antifungal activity of termite crude extracts prepared 24 h after the groups of 12 workers were exposed to the nine strains of *Metarhizium* conidia. The antifungal activity of workers also did not differ between termites exposed to either *M. brunneum* or *M. robertsii* conidia. This suggests that differential priming of the innate immune system by the different strains did not account for the observed differences in survivorship between the treatments. *Metarhizium* conidia have been shown to prime the innate immune system in *Reticulitermes*, and this affords greater protection from a secondary infection [24]. The variation in survivorship observed here may be largely due to the variation in social immunity and not variation in innate immune mechanisms such as the induced production of antimicrobial peptides. However, the crude extract antifungal activity results should be interpreted with caution as type II errors are more likely when accepting the null hypothesis that there is no relationship between alarm and antifungal activity. 

Alarm responses are complex in termites and include vertical oscillatory movements, as well as LOMs and can be elicited by a broad spectrum of stimuli representing physical, predatory and pathogenic threats [9,25]. The LOMs observed in the controls (Figure 2, Table S1) are likely to reflect alarm due to handling the insects. However, LOMs indicate that workers respond differently to two very closely related strains of *Metarhizium* that were recently considered to belong to the same species [19]. Both of these species are found in soil samples in close proximity to each other and to *Reticulitermes* colonies [17]. A differential response to the airborne odors of *Metarhizium* isolates has been observed in the subterranean termite *Coptotermes formosansus* [21] and the mound-building termite *Macrotermes michaelsensi* [20]. Both these species avoided more virulent strains and in *C. formosansus*, worker allogrooming appeared to be elevated in response to more virulent strains whose conidia were more effective at adhering to the cuticle.

## 5. Conclusions

Pathogen alarm behavior is positively correlated with rapid aggregation and increased allogrooming that reduces the mortality of the *R. flavipes* workers challenged with *Metarhizium* conidia. Isolated workers that have been challenged with *Metarhizium* conidia do not display pathogenic alarm behavior, which suggests that the alarm is a social response used to communicate the presence of a pathogenic threat to nestmates in close proximity. Different *Metarhizium* strains induce different levels of this social immune response, which appears to be largely attributable to a strong protective response to *M. robertsii* isolates and a weak response to *M. brunneum* isolates. 

## Figures and Tables

**Table 1:** Partial correlations for social behaviors and mortality while controlling for the colony of origin.

Variables	Correlation	*p*-Value	Adjusted *p*-Value
alarm, HRs	−0.617	0.008	0.024 *
alarm, allogrooming	0.502	0.040	0.048 *
alarm, aggregation	0.513	0.035	0.048 *
HRs, allogrooming	−0.431	0.084	0.084
HRs, aggregation	−0.586	0.013	0.026 *
allogrooming, aggregation	0.677	0.003	0.018 *

Significance (alpha < 0.05), after *p*-values were adjusted with Benjamini and Hochberg corrections for multiple comparisons, is indicated with an asterisk. The partial correlation tests were two-tailed.

## Supplementary Materials

The following are available online at https://www.mdpi.com/2075-4450/10/8/240/s1, Table S1: Behavioral responses and mortality of *R. flavipes* workers exposed to 9 different strains of *Metarhizium*.
